# Research trends and hotspots of cognitive behavioral therapy for tinnitus: a bibliometric analysis

**DOI:** 10.3389/fnins.2025.1536224

**Published:** 2025-05-09

**Authors:** Yu He, Jiahui Liu, Hongmei Cheng, Hongkun Ye, Chongrui Li, Yahan Gao, Xinyin Xu

**Affiliations:** ^1^College of Acupuncture and Orthopedics, Hubei University of Chinese Medicine, Wuhan, China; ^2^Department of Academic Research, Hubei Provincial Hospital of Traditional Chinese Medicine, Wuhan, China; ^3^Hubei Provincial Hospital of Traditional Chinese Medicine, Wuhan, China; ^4^The First Clinical College, Hubei University of Chinese Medicine, Wuhan, China; ^5^Hubei Shizhen Laboratory, Wuhan, China

**Keywords:** cognitive behavioral therapy, tinnitus, bibliometric analysis, trends, hotspots

## Abstract

**Background:**

Tinnitus, defined as the perception of sound without an external auditory stimulus, affects millions worldwide, significantly diminishing their quality of life. Cognitive behavioral therapy (CBT) is the most evidence-based treatment for tinnitus management, proven effective in reducing tinnitus-related distress and severity. This study aims to evaluate the current status, emerging trends, and research hotspots in CBT for tinnitus using bibliometric methods, offering valuable insights for future studies in this field.

**Methods:**

Publications related to CBT for tinnitus were retrieved from the Web of Science Core Collection (WoSCC) database from 1985 to 2024. Bibliometric analysis and visualization were performed using VOSviewer, CiteSpace, and the R package “bibliometrix.”

**Results:**

A total of 209 publications were included in this study. Research on CBT for tinnitus has shown a steady upward trend. Sweden, the United Kingdom and the United States have made notable contributions to this field. Linköping University and Karolinska Institute are the leading research institutions. Gerhard Andersson is the most prolific author and ranks first in citation frequency. The most prolific journal is the American Journal of Audiology, while Ear and Hearing is the most frequently co-cited journal. The highly cited references primarily encompass clinical trials, systematic reviews, and meta-analyses that focus on cognitive-behavioral therapy interventions. Recent keyword trends highlight topics such as “mindfulness,” “acceptance and commitment therapy,” and “internet-based interventions.” Addressing psychological comorbidities of tinnitus, including depression and anxiety, is identified as a future research focus.

**Conclusion:**

This bibliometric analysis provides a comprehensive overview of the research landscape for CBT in tinnitus management. Current research emphasizes various CBT modalities to treat psychological comorbidities associated with tinnitus. Future studies should prioritize high-quality research to confirm the long-term efficacy of CBT in tinnitus management.

## 1 Introduction

Tinnitus is an auditory perception without external sound stimulus (Piccirillo et al., 2020). It is estimated that the global prevalence of tinnitus among adults is approximately 14%, with about 2% of adults suffering from severe forms (Jarach et al., 2022). Over 120 million people worldwide are severely affected, making tinnitus a prominent global health concern (Jarach et al., 2022). Although the prevalence increases with age, tinnitus can occur at any age (Oosterloo et al., 2021). One of the primary risk factors for tinnitus is noise exposure. It disrupts the synaptic connections between cochlear hair cells and auditory nerve fibers (ANFs) through mechanical damage or metabolic overload, leading to cochlear synaptopathy and reduced auditory input to the brain (Shore et al., 2016). This peripheral damage can trigger maladaptive neuroplasticity in the central auditory system, characterized by hyperactivity in the dorsal cochlear nucleus and auditory cortex, involving increased spontaneous firing rates (SFR) and enhanced neural synchrony, ultimately resulting in the phantom perception of sound (Eggermont and Roberts, 2004; Shore and Wu, 2019). Demographic shifts and rising exposure to occupational and recreational noise suggest that the incidence of tinnitus will continue to grow (Roberts et al., 2010). Tinnitus extends beyond “ringing in the ears,” often accompanied by hyperacusis, hearing difficulties, sleep disorders, anxiety, depression, irritability, and concentration issues, all of which diminish patients’ quality of life (Langguth, 2011). Economically, tinnitus management imposes a significant financial burden on healthcare systems, patients, and society (Trochidis et al., 2021). It is estimated that in 2017, tinnitus treatment in the UK incurred a total annual healthcare cost of approximately £750 million (Stockdale et al., 2017).

Various therapeutic approaches are available for tinnitus, including pharmacological treatments, sound-based therapies, and psychological interventions. However, due to the high heterogeneity and complexity of tinnitus, these treatments have yet to eradicate tinnitus symptoms, and their efficacy is often unsatisfactory for patients. Treatments generally fall into two categories: those targeting tinnitus perception (e.g., repetitive transcranial magnetic stimulation and sound therapy) (Lefaucheur et al., 2014; Sereda et al., 2018) and those addressing the emotional and psychological responses to tinnitus, such as cognitive-behavioral therapy (CBT), relaxation techniques, and stress management (Andersson and Lyttkens, 1999; Andersson, 2002).

CBT, a psychological intervention widely used for conditions like depression and anxiety, has gained particular recognition in tinnitus management (Jun and Park, 2013). It aims to reduces the negative emotional responses to tinnitus and helps patients gradually adapt to the sound of tinnitus through psychological education, cognitive restructuring, and behavioral modification. Currently, CBT is the only intervention strongly recommended in clinical practice guidelines for tinnitus (Tunkel et al., 2014; Cima et al., 2019; Ogawa et al., 2020), and it has been proven to significantly reduce tinnitus-related distress, anxiety, and depression, and improve the quality of life for tinnitus patients (Cima et al., 2014). Over the past few decades, CBT has evolved significantly—from the “first wave” of Behavioral Therapy (BT), which focused primarily on behavioral correction, to the “second wave” of Cognitive Therapy (CT), which emphasized cognitive restructuring, and eventually to the “third wave” in the early 21st century. This latest wave integrates principles of acceptance and mindfulness, with Acceptance and Commitment Therapy (ACT) and Mindfulness-Based Cognitive Therapy (MBCT) being prominent representatives (Thoma et al., 2015; Hayes, 2016). In 2014, McKenna et al. proposed a cognitive-behavioral model of tinnitus, suggesting that negative cognitions trigger attentional focus and emotional deterioration, which in turn amplify the subjective distress associated with tinnitus, creating a vicious cycle (McKenna et al., 2014). In 2016, Ghodratitoostani et al. introduced a neurofunctional model that highlighted the role of cognitive-emotional valuation in tinnitus-related distress (Ghodratitoostani et al., 2016), further enriching the theoretical framework of CBT-t (CBT for tinnitus). As the field continues to develop, CBT interventions for tinnitus have become increasingly diverse. In addition to traditional face-to-face individual therapy, new modalities such as internet- or app-based remote psychotherapy, group-based CBT, and integrative programs combining relaxation training, biofeedback, and other therapeutic strategies have emerged. These innovations not only enhance accessibility and flexibility of care, but also offer more personalized treatment options for patients with varying needs.

With the growing global attention to tinnitus and the increasing endorsement of CBT in clinical guidelines, research on its clinical application has expanded accordingly. However, to date, no study has provided a comprehensive overview of the research landscape, trends, and emerging hotspots in this field.

Bibliometrics is a quantitative research method that uses mathematical and statistical tools to analyze publication patterns and trends in scientific literature (Ninkov et al., 2022). By constructing a knowledge map of academic publications, researchers can identify key countries, journals, institutions, and authors, as well as uncover significant references and keywords. This approach also reveals the current state, development trends, and emerging hotspots in the field. Indeed, several studies have investigated the research trends and hotspots in the field of tinnitus treatment. For instance, Ye et al. (2022) conducted a systematic evaluation of global research trends and emerging topics in tinnitus treatment in the 21st century, highlighting cognitive behavioral therapy (CBT) as a notable research focus, although without providing a deeper exploration of this aspect. Similarly, Hu et al. (2023) adopted an innovative approach by integrating bibliometric analysis with product comparison methods to examine the research trends, product characteristics, and translational outcomes of mobile health (mHealth) applications in tinnitus management. However, their study primarily concentrated on digital therapeutics rather than psychological intervention strategies. While these studies have contributed to our understanding of tinnitus treatment, there remains a lack of bibliometric research specifically focusing on the application of CBT in this context. Therefore, the present study employs a rigorous knowledge mapping methodology and visualization techniques to systematically characterize the current landscape, developmental trajectory, and research hotspots of CBT in the treatment of tinnitus, addressing a critical gap in the literature. This work aims to advance tinnitus management, benefiting both researchers and clinicians.

## 2 Methods

### 2.1 Data sources and search strategies

**Figure 1:**
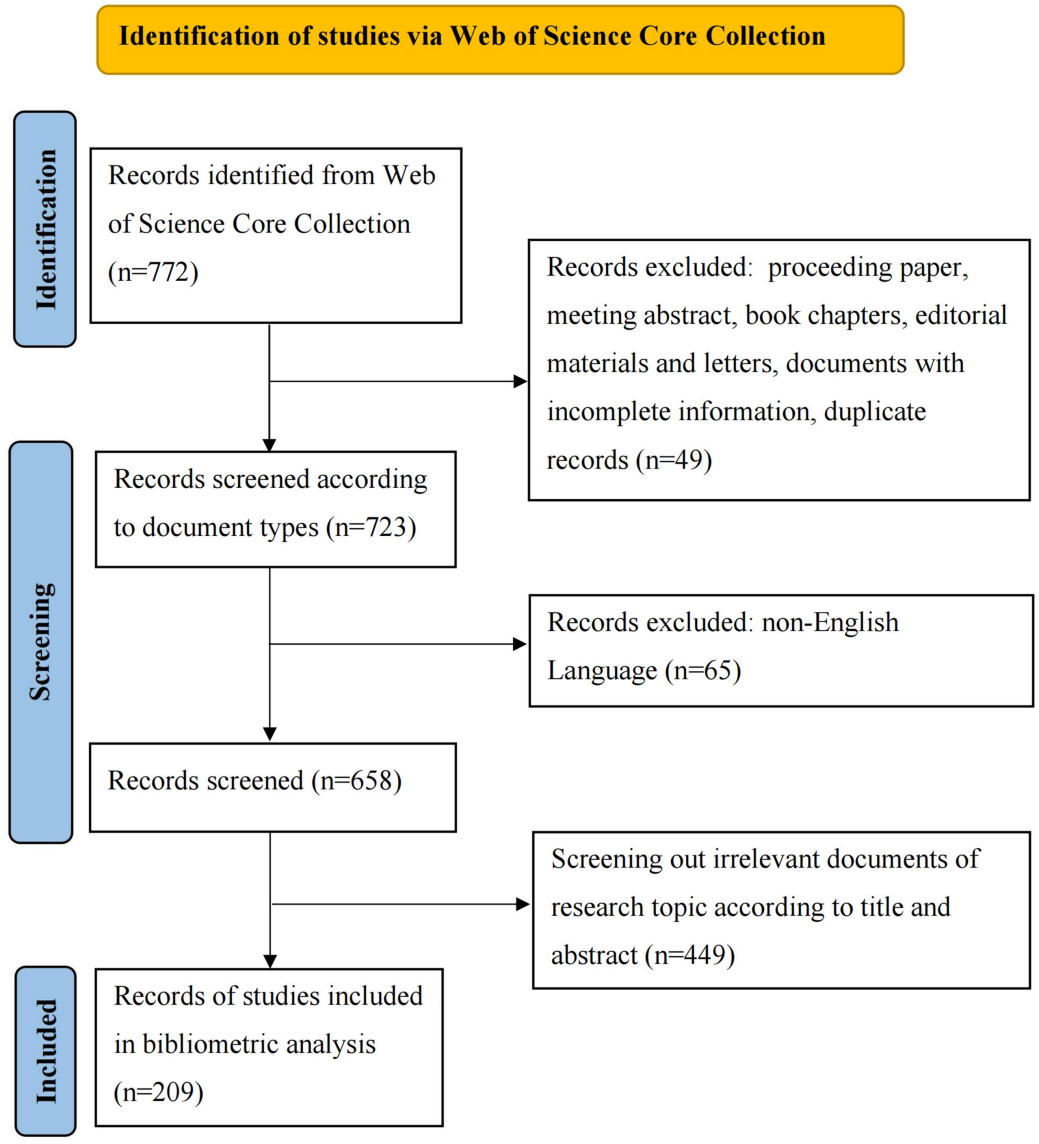
Screening flow chart.

The bibliometrics data of this study came from the Web of Science Core Collection (WosCC) database. WoSCC is widely regarded as the preferred database for bibliometric analysis due to its rigorous journal inclusion standards, comprehensive citation indexes, and high-quality literature resources (Thompson and Walker, 2015). The publication time span was from January 1, 1985 to August 2, 2024. In order to prevent data bias from database changes, the search was finished on August 2, 2024. The main search terms were “tinnitus,” “cognitive behavioral therapy (CBT)” and other forms of CBT including internet-based CBT (iCBT), group-based CBT (gCBT), Acceptance and Commitment Therapy (ACT), mindfulness, mindfulness-based CT (MBCT). The detailed search strategy is shown in Table 1. To ensure the quality and relevance of the research literature, a manual screening process is necessary. Therefore, the authors (Y.H. and J.H.L.) independently screened the titles and abstracts to identify available studies based on the following criteria. The inclusion criteria encompassed: (1) publications with titles or abstracts explicitly mentioning CBT and tinnitus as the primary research topics. (2) language was limited to English. The exclusion criteria comprised: (1) proceeding paper, meeting abstract, book chapters, editorial materials and letters; (2) literature with duplicated content; and (3) papers with incomplete information (e.g., author details, journal name, and keywords). Ultimately, a total of 209 publications highly relevant to the research theme of CBT and tinnitus were included. The complete screening procedure is demonstrated in Figure 1.

**Table 1:** Topic search query.

Set	Search query
#1	TS = (Tinnitus)
#2	TS = ((cogniti* or behavio* or relaxation or acceptance or commitment or adaptation or meditation) near/6 (therap* or intervention* or approach* or psychotherap* or training or treatment* or technique* or counseling))
#3	TS = (CBT or iCBT or GCBT or ACT or iACT or mindfulness or MBSR or MBTSR or MBCT)
#4	#2 OR #3
#5	#1 AND #4

### 2.2 Data analysis and visualization

Statistical calculations and graphics were done using VOSviewer, CiteSpace, R-bibliometric and Microsoft Office Excel 2016. The data collected from WoSCC was used to create bibliometric maps and VOSviewer, CiteSpace, Bibliometrix were used to collate the relevant literature in the field and visualize the results to analyze the potential information, respectively. Microsoft Office Excel 2016 was used to draw the statistical graphs.

## 3 Result

### 3.1 Analysis of publication trend

**Figure 2:**
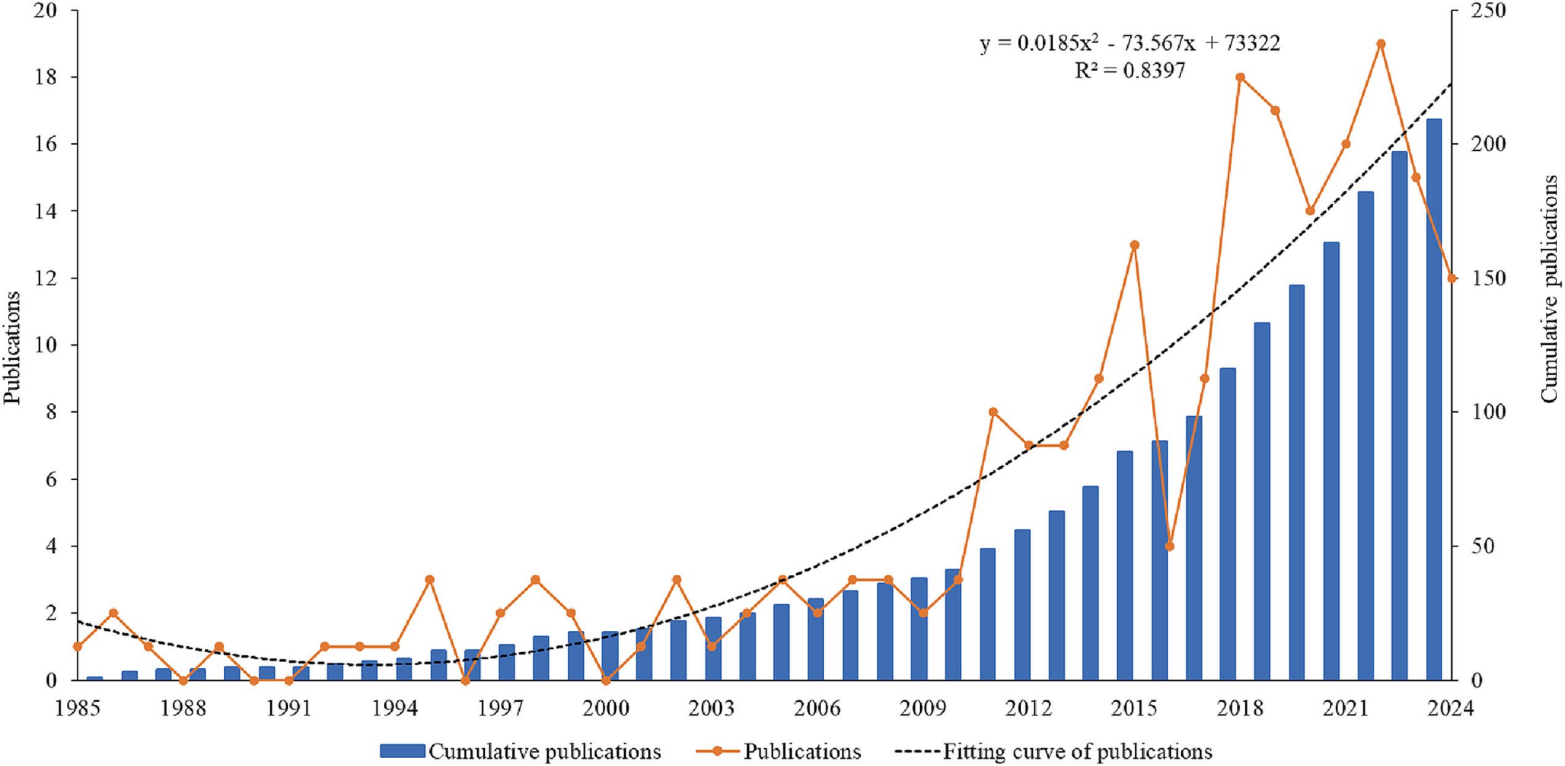
Annual publication growth trend of cognitive behavioral therapy for tinnitus from 1985 to 2024.

The variation in annual publication volume reflects the level of academic attention devoted to a specific research field (Liu et al., 2024). By plotting the temporal distribution curve of annual publication volume, it is possible to preliminarily evaluate the current status of the field, as well as forecast its potential development trends. As depicted in Figure 2, there were 209 cumulative publications on cognitive behavioral therapy for tinnitus from 1985 to 2024, with the number of annual publications showing large fluctuations. Notably, from 2016 to 2018, the number of publications shapely increased and reached a peak of 19 in 2022. The number of annual publications exceeded 10 in the last 7 years. Moreover, the curve of the annual number of publications fits a quadratic function, with a goodness-of-fit R^2^ of 0.8397, indicating that the number of publications is expected to increase even more rapidly in the future.

### 3.2 Analysis of countries/region and institutions

**Figure 3:**
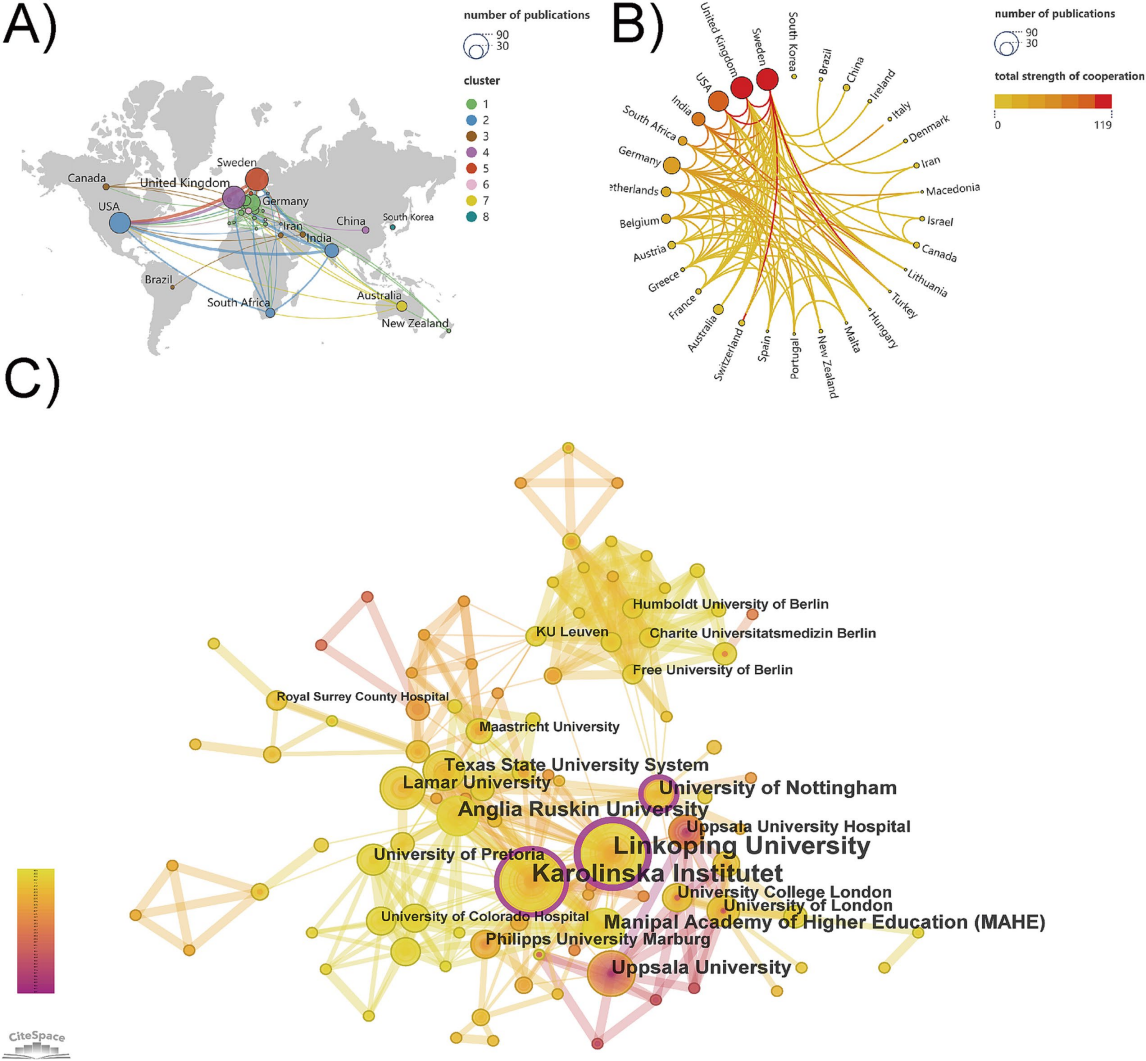
The analysis of countries/regions and institutions. **(A)** Map of the geographic location of countries. The size of the node represents the number of national publications. **(B)** Chord chart of the strength of countries cooperation. The deeper the color of the curved connecting line, the stronger the cooperation strength it represents. **(C)** The co-occurrence and collaboration map of research institutions. Nodes in the purple round indicate high betweenness centrality (≥0.1).

A total of 30 countries/regions and 313 institutions were involved in cognitive behavioral therapy for tinnitus research. As displayed in Figure 3A, VOS clustering indicates the division of the inter-country cooperation networks into 8 clusters of which 7 are cooperative network clusters, while South Korea do not cooperate with other countries (cluster #8). The thickness of the line between countries indicates the strength of cooperation. The map shows three distinct geographical advantages with research clusters led by European countries (cluster #1, cluster #4, cluster #5), the American continental research cluster (cluster#2, cluster#3), and the Oceanian research cluster (cluster #7). Table 2 presents the top 10 countries/regions in terms of publication count, along with their citation frequency and total link strength. Sweden and the United Kingdom were the leading countries in terms of publications, the same with 68, followed by the USA (58), Germany (40), and other countries with less than 40 publications. Additionally, Sweden also ranks first in citation counts and total link strength, indicating the significant influence of Sweden in this research area. Sweden, the UK and the USA as the core cluster, showing closer cooperation (Figure 3B).

**Table 2:** The top 10 productive countries concerning cognitive behavioral therapy for tinnitus.

Rank	Country	Documents	Citations	Total link strength
1	Sweden	68	3,602	119
2	United Kingdom	68	2029	109
3	USA	58	821	98
4	Germany	40	2,183	46
5	India	25	372	84
6	Australia	14	230	11
7	Netherlands	13	1,442	21
8	Belgium	12	479	18
9	South Africa	11	54	47
10	Austria	8	87	15

In the analysis of institutional cooperation, Table 3 presents the top 10 research institutions regarding the number of publications, with Linkoping University from Sweden leading with 52 publications, followed by Karolinska Institutet from Sweden with 48 publications, and Anglia Ruskin University from the United Kingdom with 23 publications. Karolinska Institutet and Linkoping University reflecting their research strength and importance. In addition, the centrality of Linkoping University, the University of Nottingham, and Karolinska Institutet is greater than 0.1, indicating that these three institutions play a significant role in institutional collaboration (Figure 3C).

**Table 3:** The top 10 contributing institutions related to cognitive behavioral therapy for tinnitus.

Rank	Institution	Count	Centrality	Year	Country
1	Linkoping University	52	0.21	2005	Sweden
2	Karolinska Institutet	48	0.12	2009	Sweden
3	Anglia Ruskin University	23	0.08	2015	United Kingdom
4	Manipal Academy of Higher Education (MAHE)	20	0.01	2017	India
5	University of Nottingham	18	0.18	2011	United Kingdom
6	Uppsala University	16	0.03	1987	Sweden
7	Lamar University	15	0.00	2015	USA
8	Texas State University System	14	0.00	2015	USA
9	Uppsala University Hospital	12	0.02	1987	Sweden
10	University of London	11	0.04	1992	United Kingdom

### 3.3 Analysis of co-authors and co-cited authors

**Figure 4:**
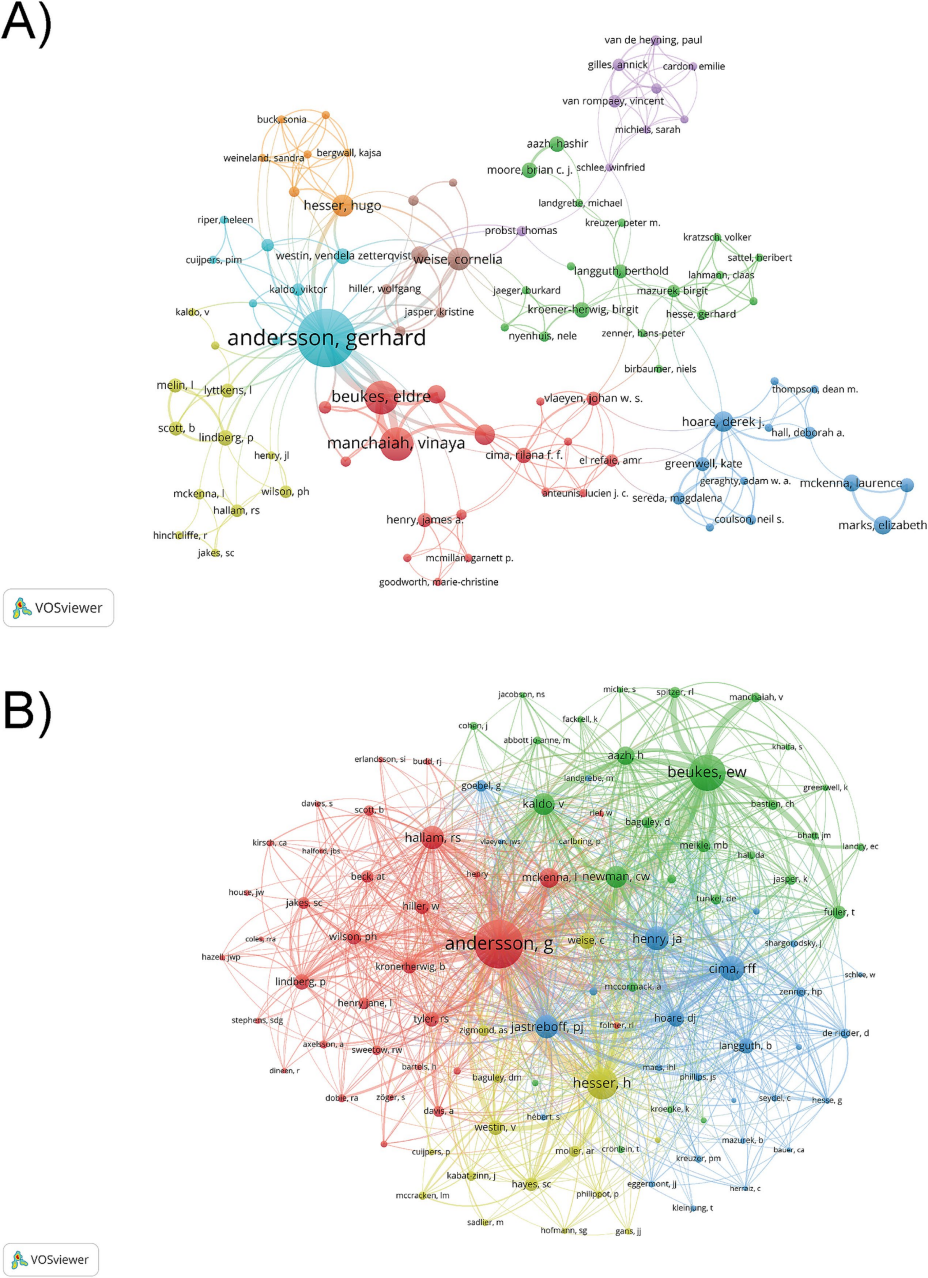
The analysis of authors. **(A)** Network map of co-authors collaboration. The size of the nodes represents the number of publications by the authors, the links within the network represent collaborations between authors, and the thickness of the links indicates the strength of these collaborations. **(B)** Network map of co-cited authors collaboration. The size of the nodes represents the citations frequency of authors.

We utilized VOSviewer to analyze 130 authors who had published at least two papers. The author with the most published papers is Gerhard Andersson (60) from Linkoping University, followed by Beukes, Eldre W. (23) from Anglia Ruskin University, and Manchaiah, Vinaya (23) from University of Colorado-Anschutz Medical Campus (Table 4). The three authors show a close collaboration relationship in this field (Figure 4A). It is noteworthy that Gerhard Andersson stands out as both the most frequently co-cited and cited author within this domain, indicating a high level of academic attention of his contributions. He plays a central role in the collaborative network and is the most critical contributor to the research on cognitive behavioral therapy for tinnitus. The co-citation of authors refers to when two or more authors are cited by other publications at the same time, these authors form a co-citation relationship. When the number of co-citations is higher, their academic research is more similar, and the analysis reflects their research strength. We have conducted an analysis of authors who have been co-cited at least 15 times, and 104 authors met this criterion. As shown in Figure 4B, the authors were mainly divided into 4 clusters: Gerhard Andersson, Richard S. Hallam etc. (red), Eldre W. Beukes, Viktor Kaldo etc. (green), Rilana F. F. Cima, J. A. Henry, etc. (blue), Hugo Hesser, Vera Westin, etc. (yellow). The high citation counts of these authors suggest that their research has been widely acknowledged and utilized by other researchers in the field, highlighting the significant influence of their publications.

**Table 4:** The top 10 co-authors and co-cited authors.

Rank	Co-authors	Documents	H-index	Citations	Total link strength	Co-cited authors	Citations	Total link strength
1	Andersson, Gerhard	60	27	3,154	160	Andersson, Gerhard	423	11,090
2	Beukes, Eldre W.	23	12	344	69	Beukes, E.W.	266	6,163
3	Manchaiah, Vinaya	23	12	344	69	Hesser, Hugo	213	5,673
4	Weise, Cornelia	12	11	585	41	Cima, R.F.F.	152	4,905
5	Hesser, Hugo	11	9	723	35	Henry, J.A.	145	4,259
6	Baguley, David M	10	9	465	42	Hallam, R.S.	139	3,912
7	Hoare, Derek J	10	7	501	31	Jastreboff, P.J.	138	5,106
8	Allen, Peter M	8	7	242	32	Kaldo, Viktor	125	3,636
9	Marks, Elizabeth	8	6	111	11	Newman, CW	121	3,333
10	Kleinstaeuber, Maria	7	7	223	27	Mckenna, L	103	3,028

### 3.4 Analysis of journals

**Figure 5:**
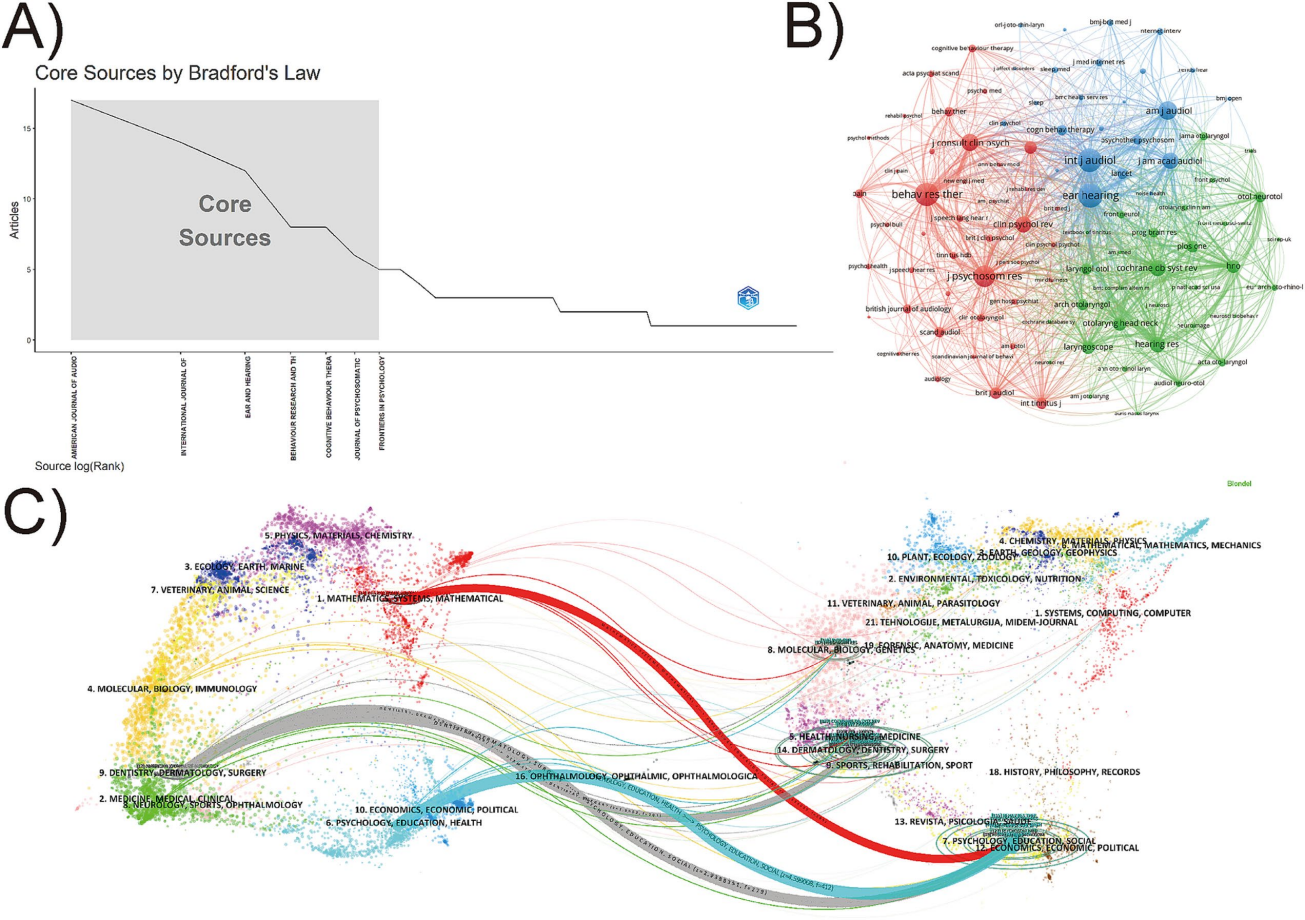
The analysis of academic journals. **(A)** Bradford’ s Law applied to academic journals, and the shaded gray area in the figure illustrates the core journals in the field, ranked in descending order of the number of articles. **(B)** Network visualization of co-cited journals. The size of the node indicates the citations frequency of journals. **(C)** The dual-map overlay of journals.

Bradford’s Law delineates the distribution regularities of scholarly articles in different journal. According to the law, we identified 7 core journals in the field of cognitive behavioral therapy for tinnitus research (Figure 5A). The journal with the most publications is American Journal of Audiology (17 publications, IF:1.4, JCR: Q2), followed by the International Journal of Audiology (14 publications, IF:1.8, JCR: Q2), Ear and Hearing (12 publications, IF:2.6, JCR: Q1), and several other journals with fewer than 10 publications (Table 5). Among the top 10 co-cited academic journals, Ear and Hearing, Behaviour Research and Therapy, and International Journal of Audiology each had citation frequencies exceeding 300, indicating their significant academic impact and recognition in this research field. Among the top 10 journals and co-cited journals, most were JCR Q1 and Q2, but their impact factor (IF) is less than 5.

**Table 5:** The top 10 academic journals and co-cited journals on cognitive behavioral therapy for tinnitus.

Rank	Journals	Documents	JCR	IF	Co-cited journals	Citations	JCR	IF
1	American Journal of Audiology	17	Q2	1.4	Ear and Hearing	325	Q1	2.6
2	International Journal of Audiology	14	Q2	1.8	Behaviour Research and Therapy	315	Q1	4.2
3	Ear and Hearing	12	Q1	2.6	International Journal of Audiology	314	Q2	1.8
4	Behaviour Research and Therapy	8	Q1	4.2	Journal of Psychosomatic Research	276	Q2	3.5
5	Cognitive Behaviour Therapy	8	Q1	4.3	American Journal of Audiology	220	Q2	1.4
6	Journal of Psychosomatic Research	6	Q2	3.5	Journal of Consulting and Clinical Psychology	202	Q1	4.5
7	Frontiers in Psychology	5	Q2	2.7	Cochrane Database of Systematic Reviews	187	Q1	8.8
8	Journal of Laryngology and Otology	5	Q3	1.1	Journal of the American Academy of Audiology	185	Q3	1.0
9	Trials	4	Q3	2.0	Clinical Psychology Review	184	Q1	13.7
10	Bmj Open	3	Q1	2.4	Hearing Research	174	Q1	2.5

Figure 5B shows an analysis of 124 journals that have been co-cited more than 15 times. These journals have been categorized into three clusters based on their co-citation frequency, indicating that they tend to focus on similar research directions. The red cluster is mainly focused on Psychiatry and Clinical Psychology (Behaviour Research and Therapy, Journal of Psychosomatic Research, Journal of Consulting and Clinical Psychology, etc.), the blue cluster is mainly in the field of Audiology (Ear and Hearing, International Journal of Audiology and American Journal of Audiology, etc.), the green cluster focused on medicine and Otolaryngology (Cochrane Database of Systematic Reviews, Hearing Research, and Otolaryngology Head and Neck Surgery, etc.).

Figure 5C presents a dual-map overlay of journals, reflecting the flow of knowledge between the citing literature and the cited literature. The left half of shows the distribution of the disciplines of the citing literature as the current research status of cognitive behavioral therapy for tinnitus. The right half shows the disciplines of the cited literature as the research basis of cognitive behavioral therapy for tinnitus. The colored paths illustrate the relationships between citations. The horizontal axis of the ellipse indicates the number of publications in the journal. Within the cited regions, the most popular fields with the most documents covered are “DENTISTRY, DERMATOLOGY, SURGERY” and “PSYCHOLOGY, EDUCATION, SOCIAL,” the major citation trajectories originate in these two area and progress to the frontier research area of “MATHEMATICS, SYSTEMS, MATHEMATICAL,” “PSYCHOLOGY, EDUCATION, HEALTH” and “DENTISTRY, DERMATOLOGY, SURGERY.” Furthermore, when “PSYCHOLOGY, EDUCATION, HEALTH” groups were used as citing journals, the “PSYCHOLOGY, EDUCATION, SOCIAL” groups had the highest number of citations, with the highest Z value of 4.59. The blue citation trajectory emphasizing the significance and impact of this development path.

### 3.5 Analysis of co-cited references and reference bursts

**Figure 6:**
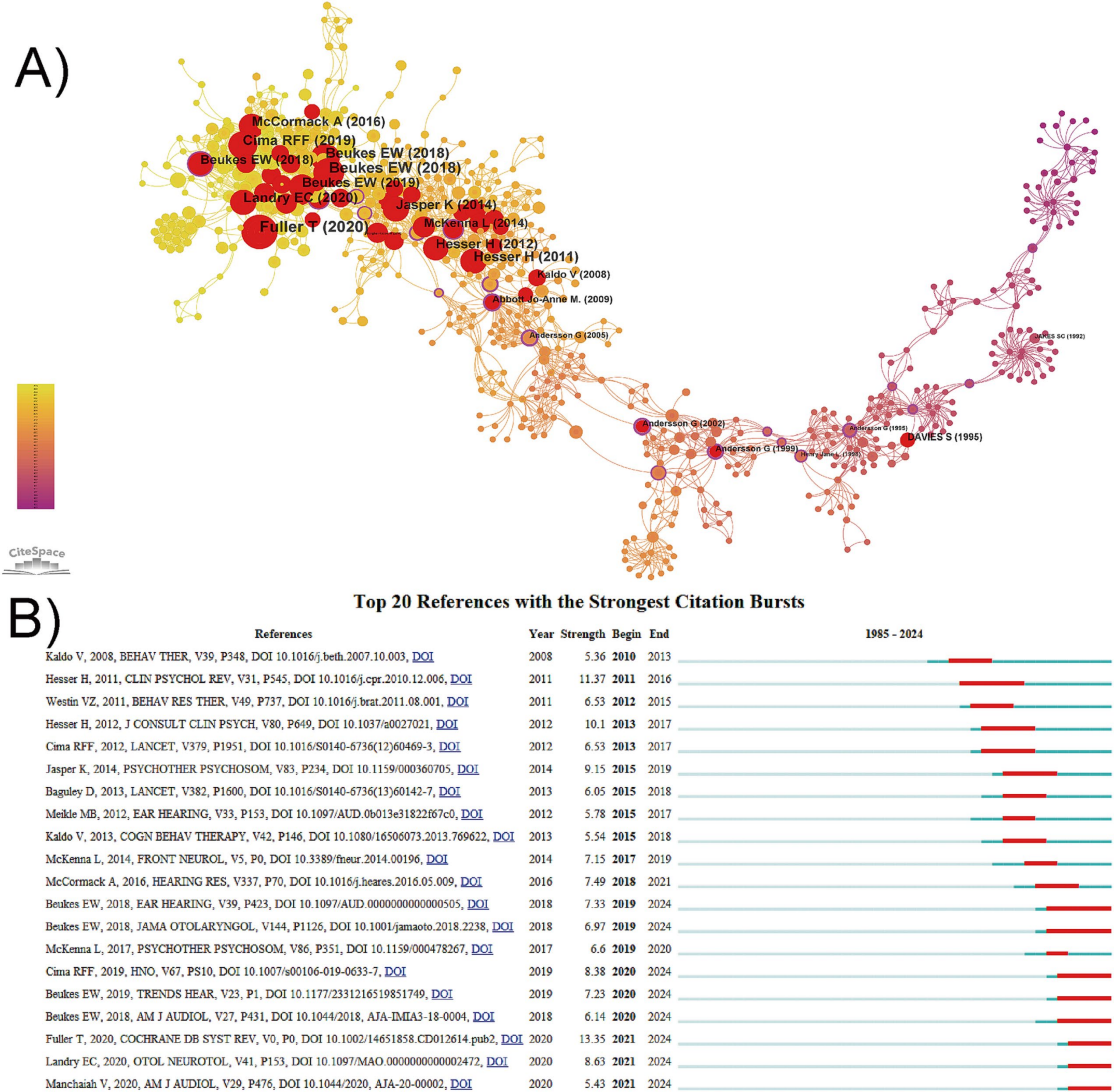
The analysis of co-citation references. **(A)** Visualization of co-citation references, where red nodes represent bursts detection. **(B)** The top 20 references with the strongest citation bursts, where red bars indicate high citations in the corresponding year.

The CiteSpace’s co-citation analysis of references is a core feature of this program. A scaling factor k = 25 was set, and 798 cited references with certain influences were extracted using g-index that allowed the identification of homogeneous clusters of literature that was cited more frequently and was associated with CBT for tinnitus research. Table 6 summarizes the top 10 most cited articles on CBT for tinnitus treatment. These articles span from 2011 to 2020, and apart from one guideline for tinnitus, the types of literature include randomized controlled trials, systematic reviews, and meta-analyses. Figure 6A shows the co-citation mapping of CBT for tinnitus. The size of the nodes represents the co-citation frequency, with larger nodes indicate higher co-citation frequencies and red nodes represent burst citations. The most cited paper (39 citations) was “Cognitive behavioral therapy for tinnitus (Fuller et al., 2020),” published in the Cochrane Database of Systematic Reviews. References with citation bursts refer to those that experience a sudden and significant increase in citation frequency within a specific period. As shown in Figure 6B, the earliest citation burst in the field of cognitive behavioral therapy for tinnitus began in 2010. The strongest burst (strength:13.35) occurred in a paper entitled “Cognitive behavioral therapy for tinnitus” published in Cochrane Database of Systematic Reviews by Fuller et al. (2020), with citation burst from 2021 to 2024. This is followed by “A systematic review and meta-analysis of randomized controlled trials of cognitive-behavioral therapy for tinnitus distress” (strength: 11.37, publication year: 2011) and “A Randomized Controlled Trial of Internet-Delivered Cognitive Behaviour Therapy and Acceptance and Commitment Therapy in the Treatment of Tinnitus” (strength: 10.1, publication year: 2012), and Hesser, Hugo is the same authors of these two papers (Hesser et al., 2011; Hesser et al., 2012). Moreover, eight references had citation bursts lasting through 2024.

**Table 6:** The top 10 co-cited references of cognitive behavioral therapy for tinnitus.

Rank	Title	First author	Journal	Co-citation frequency	Year
1	Cognitive behavioral therapy for tinnitus	Fuller, T	Cochrane Database of Systematic Reviews	39	2020
2	Audiologist-Guided Internet-Based Cognitive Behaviour Therapy for Adults With Tinnitus in the United Kingdom: A Randomized Controlled Trial	Beukes, EW	Ear and Hearing	30	2018
3	A multidisciplinary European guideline for tinnitus: diagnostics, assessment, and treatment	Cima, RFF	HNO	25	2019
4	Effectiveness of Guided Internet-Based Cognitive Behavioral Therapy vs. Face-to-Face Clinical Care for Treatment of Tinnitus: A Randomized Clinical Trial	Beukes, EW	JAMA Otolaryngology Head Neck Surgery	24	2018
5	A systematic review and meta-analysis of randomized controlled trials of cognitive-behavioral therapy for tinnitus distress	Hesser, H	Clinical Psychology Review	23	2011
6	Systematic Review and Network Meta-analysis of Cognitive and/or Behavioral Therapies (CBT) for Tinnitus	Landry, EC	Otology & Neurotology	22	2020
7	Internet-based guided self-help versus group cognitive behavioral therapy for chronic tinnitus: a randomized controlled trial	Jasper, K	Psychotherapy and Psychosomatics	21	2014
8	A randomized controlled trial of Internet-delivered cognitive behaviour therapy and acceptance and commitment therapy in the treatment of tinnitus	Hesser, H	Journal of Consulting and Clinical Psychology	20	2012
9	Internet-Based Interventions for Adults With Hearing Loss, Tinnitus, and Vestibular Disorders: A Systematic Review and Meta-Analysis	Beukes, EW	Trends in Hearing	20	2019
10	A systematic review of the reporting of tinnitus prevalence and severity	McCormack, A	Hearing Research	19	2016

### 3.6 Analysis of keyword

**Figure 7:**
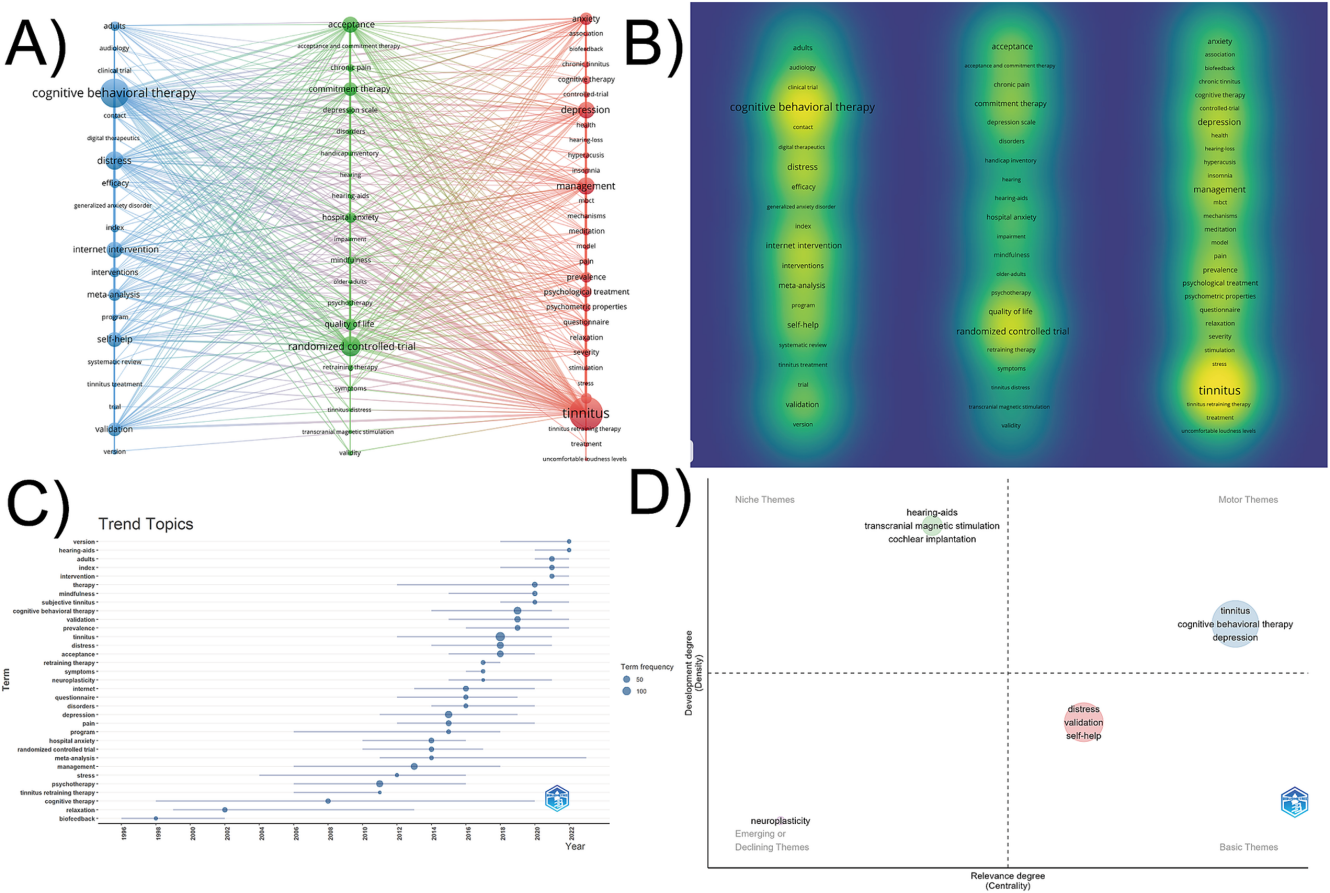
The analysis of keywords. **(A)** The co-occurrence and cluster network of keywords. **(B)** The density visualization of keywords. **(C)** Trend topics map, with the 3 keywords per year which occurred at least 5 times in the past decades. **(D)** Thematic map of cognitive behavioral therapy for tinnitus. The horizontal axis represents the centrality of the theme, indicating the strength of the links between the thematic cluster and other themes, while the vertical coordinate represents the development degree. The thematic map is divided into four quadrants: Motor themes in the first quadrant represented core themes with high centrality and maturity, niche themes in the second quadrant represented independent themes with high maturity, the third quadrant represented emerging or declining themes, and basic themes in the fourth quadrant indicated popular themes with high centrality and low maturity.

Keywords reflect the topic and research content of the literature, and are a refined summary of the research content. Analyzing the co-occurrence of keywords provides valuable insights into the focus and trends of research within a specific field. Using VOSviewer for keyword co-occurrence analysis, and classifying keywords into clusters based on their intrinsic connections and frequencies. Synonyms were merged for data consistency, such as “cognitive behavioral therapy” and “cognitive behavioral therapy,” “rct” and “randomized controlled trial,” “internet-based intervention” and “internet intervention” etc. 71 keywords were extracted by setting the keyword frequency to 5 to obtain the keyword clustering map of the research on cognitive behavioral therapy for tinnitus, and 3 clusters of representing three major themes were obtained. As shown in Figure 7A, Cluster 1 (blue cluster) where “cognitive behavioral therapy,” “distress,” “internet intervention,” “self-help” were representative keywords. It chiefly emphasized the application of cognitive behavioral therapy. Cluster 2 (green cluster) correlated with “acceptance and commitment therapy.” Representative keywords include “acceptance,” “commitment therapy,” “mindfulness,” “quality of life,” and “randomized controlled trial.” Cluster 3 (red cluster): psychological comorbidities of tinnitus. Representative keywords include “tinnitus,” “depression,” and “anxiety.” Using VOSviewer to visualize keyword density (Figure 7B), with colors closer to yellow indicating a higher frequency of keyword occurrence. As shown in Table 7, the frequently occurring keywords are “tinnitus (131),” “cognitive behavioral therapy (105),” “randomized controlled trial (62),” “distress (54),” “management (47),” “depression (45)” and “internet intervention (44).”

**Table 7:** The top 15 keywords in the field of cognitive behavioral therapy for tinnitus.

Rank	Keyword	Occurrences
1	tinnitus	131
2	cognitive behavioral therapy	105
3	randomized controlled trial	62
4	distress	54
5	management	47
6	depression	45
7	internet intervention	44
8	acceptance	42
9	self-help	38
10	validation	35
11	commitment therapy	34
12	meta-analysis	29
13	anxiety	29
14	quality of life	28
15	psychological treatment	25

To identify the research hotspots and future trend topics in the field of cognitive behavioral therapy for tinnitus, we conducted an in-depth analysis by integrating trend topic map and thematic map. Specifically, several trend topics (Figure 7C), such as “version,” “hearing-aids,” “adults,” “index,” “intervention,” “therapy,” and “mindfulness” gained considerable traction over the past 3 years. As shown in Figure 7D, the blue cluster (“tinnitus,” “cognitive behavioral therapy,” and “depression”) is located in the first quadrant, representing the core topics in this field, with high development degree and high centrality. This indicates that CBT for treating tinnitus and its psychological comorbidities has reached a relatively mature stage. The green cluster (“hearing-aids,” “transcranial magnetic stimulation,” and “cochlear implantation”) is situated in the second quadrant, representing independent topics with a high degree of development but low centrality, which representing other treatments for tinnitus or hearing loss. The red cluster (“distress,” “validation,” and “self-help”) is located in the fourth quadrant, representing fundamental topics with high centrality but low development degree, associated with tinnitus management. Additionally, “neuroplasticity” is an emerging or declining theme related to the mechanisms of tinnitus.

## 4 Discussion

### 4.1 General information

A total of 209 publications on cognitive behavioral therapy for tinnitus research were included in the analysis from 1985 to 2024 by searching the WoSCC. In the past four decades, although the number of publications on cognitive behavioral therapy for tinnitus has been limited, there has been a growing trend in research output. There were two significant spikes in publication volume within this field in 2018 and 2022, which indicated that more attention had been paid to this field.

The analysis of academic output and collaboration reveals that the top 10 countries and institutions are primarily from Europe, North America, and Asia, with all being developed countries except India and South Africa. Sweden, the UK, and the USA are leading in this field. This highlights the strong correlation between academic productivity and economic strength, emphasizing the dominance of developed countries and their research institutions in this area. Interestingly, South Korea has formed a relatively isolated research cluster. An analysis of the three included South Korean studies (Lee et al., 2004; Lee and Jung, 2023; Jeong et al., 2024) revealed that all co-authors were affiliated with domestic institutions, with no participation in international collaborations. This phenomenon may be attributed to divergent regional research priorities or explorations of culturally adaptive research tailored to local contexts. Such a relatively closed research ecosystem may limit the diversified development of research perspectives and the sharing of cutting-edge technologies, further undermining the global influence of research outcomes and the progress of evidence-based practices. Therefore, in the field of psychological interventions for tinnitus, which highly relies on interdisciplinary integration, it is crucial to break regional academic boundaries and establish multinational collaboration networks.

For the author contributions, Gerhard Andersson from Linkoping University in Sweden stands out, leading in publication output, citation frequency, and H-index. He and his research team developed a self-help internet-based CBT for tinnitus, initially validating its efficacy and feasibility through a randomized controlled trial, thereby pioneering a new model of telepsychological treatment (Andersson et al., 2002). Furthermore, his research team evaluated the acceptability and feasibility of iCBT for tinnitus in the UK and US through cross-cultural adaptability (e.g., language localization) and technical optimization (e.g., secure platform encryption), providing valuable experience for the global implementation of iCBT (Beukes et al., 2016; Manchaiah et al., 2020). From a broader interdisciplinary perspective, tinnitus research has shown a trend of cross-domain integration. In artificial intelligence applications, data mining techniques and machine learning models have been employed to predict treatment outcomes of iCBT for tinnitus (Rodrigo et al., 2021). Similarly, large language models (e.g., GPT-2, Google T5, Flan-T5) have been utilized to analyze CBT session text data of tinnitus patients, enabling precise prediction of therapeutic efficacy and optimization of clinical case management through digital tools (Jeong et al., 2024). Notably, the Shabestari team applied explainable artificial intelligence methods to analyze resting-state electroencephalography (EEG) datasets, revealing critical neural signatures associated with brief acoustic tinnitus suppression, thereby providing significant insights into the neural mechanisms of tinnitus and personalized treatment strategies (Shabestari et al., 2025). In the field of behavioral sciences, Ecological momentary assessment (EMA) has been introduced as a dynamic monitoring tool for tracking tinnitus symptom fluctuations (Engelke et al., 2023). It has been found that smartphone-based EMA can efficiently capture real-time symptom dynamics and identify factors influencing symptoms, enhancing the accuracy of tinnitus assessments (Wilson et al., 2015). Moreover, immersive virtual reality (VR) technology has also been applied to tinnitus intervention. In a study conducted by Park’s team (Park et al., 2022), customized VR protocols were administered to chronic subjective tinnitus patients, with therapeutic outcomes evaluated through combining EEG spectral analysis with psychometric questionnaires. The findings suggest that VR may alleviate tinnitus distress by modulating cognitive and emotional functions of the prefrontal-limbic network. Future research directions should focus on systematic integration of these multidisciplinary approaches to address tinnitus heterogeneity challenges and promote advancements in tinnitus management.

American Journal of Audiology is the journal with the highest number of publications. Three journals were cited more than 300 times, with Ear and Hearing (IF: 2.6, JCR: Q1) having the most citations, indicating the journal’s academic status and influence in tinnitus-related treatment research. However, the proportion of articles published in high-impact journals in the field of CBT for tinnitus is relatively low, which may be attributed to the interdisciplinary nature of the field and its current stage of development.

Co-citation of references can be regarded as a knowledge base for a specific research field. The article with the highest citation count and the strongest burst strength is “Cognitive behavioral therapy for tinnitus” published by Fuller, Thomas in the Cochrane Database of Systematic Reviews in 2020 (Fuller et al., 2020). This review, through the analysis of 28 randomized controlled trials, found that compared to no treatment or waiting list control for tinnitus, CBT significantly improved the severity of tinnitus and may to a lesser extent reduce depressive symptoms. Compared to audiological care and tinnitus retraining therapy, CBT is more effective in improving quality of life. Furthermore, CBT rarely leads to any adverse effects, but further research is needed to verify its long-term efficacy (Fuller et al., 2020). A randomized controlled trial was the first to compare the efficacy of internet-delivered ACT with traditional CBT for tinnitus. The results showed that ACT was equally effective as traditional CBT in reducing tinnitus severity, with treatment effects remaining stable at one-year follow-up. Meanwhile, ACT demonstrated significant advantages in alleviating comorbid depression and perceived stress. This study confirmed the feasibility of internet-based psychological interventions in tinnitus management and suggested that ACT could serve as a viable alternative to traditional CBT (Hesser et al., 2012). Future research should continue to refine these interventions, assess their long-term outcomes, and explore their integration into routine clinical practice.

### 4.2 Trends and hotspots

#### 4.2.1 Tinnitus and comorbid depression and anxiety: CBT interventions

In recent years, tinnitus and its psychological comorbidities, particularly depression and anxiety, have garnered increasing attention from researchers. A comprehensive analysis of the data presented in Figures 7A,D reveals that CBT for the treatment of tinnitus accompanied by depressive and anxious symptoms has emerged as a mature and highly regarded topic within this field, currently experiencing robust development. Anxiety and depression, as common psychological comorbidities in tinnitus patients, are prevalent among clinical tinnitus populations, with incidence rates maintaining a high level (Gül et al., 2015; Durai and Searchfield, 2016). Reports indicate that the prevalence of major depression among tinnitus patients can reach as high as 60–78% (Sullivan et al., 1988), and the lifetime prevalence of tinnitus patients with comorbid anxiety disorders is 45% (Pattyn et al., 2016). A population-based cohort study have demonstrated a significant correlation between tinnitus and depression, as well as anxiety (Hackenberg et al., 2023). However, it is currently not fully established whether depression and anxiety are risk factors or consequences of tinnitus (McCormack et al., 2015; Hébert, 2021). The higher the severity of tinnitus, the greater the likelihood of comorbidity (Langguth et al., 2011; Beukes et al., 2021). One study has shown that the severity of tinnitus correlates with the severity of depression and anxiety, highlighting the crucial importance of considering depressive and anxiety disorders when treating tinnitus patients (Zöger et al., 2006). CBT, as a form of psychotherapy originally developed for the treatment of depression and anxiety, has been proven effective in alleviating tinnitus-related distress. For example, Beukes et al. conducted a randomized controlled trial utilizing iCBT as an intervention, which demonstrated that the experimental group experienced significant reductions in tinnitus distress and comorbid symptoms, as well as improved quality of life. This study provides evidence for the effectiveness of CBT in treating tinnitus (Beukes et al., 2018b).

#### 4.2.2 Factors and strategies for the slow progress of CBT-t

Based on previous research results and the findings of this study, although there is evidence supporting CBT’s efficacy in reducing tinnitus-related distress, the development of CBT in the field of tinnitus treatment is still relatively slow. This phenomenon can be attributed to several main factors: (1) Tinnitus is a complex disorder with high heterogeneity (Cederroth et al., 2019), displaying significant differences in clinical presentation, symptom severity, and comorbidities such as anxiety and depression among different patients. Therefore, treatment of tinnitus requires personalized multidisciplinary approaches integrating expertise from otolaryngology, audiology, psychology, and psychiatry to meet individual patient needs. (2) Despite the potential advantages of multidisciplinary approaches, collaboration between disciplines faces numerous obstacles in clinical practice, limiting its widespread promotion and application. Currently, most CBT programs are guided by psychologists. Although studies have confirmed the effectiveness of audiologist-provided CBT programs for tinnitus (Aazh and Moore, 2018), these programs differ in procedural details and research perspectives, and their equivalence to psychologist-provided CBT needs further verification (Burke and El Refaie, 2024). Additionally, the ability of audiologists to manage significant comorbid mental health issues may be limited (Henry et al., 2022), possibly due to specialization in medical fields and differing emphases on specialized knowledge. (3) CBT requires specially trained psychotherapists to implement, but such professionals are currently scarce and service resources are limited. Within the UK healthcare system, for instance, tinnitus patients encounter dual obstacles in accessing psychological interventions: a shortage of mental health professionals and inefficient referral pathways to psychological services (Gander et al., 2011; McFerran et al., 2018). (4) Patients’ cognitive biases and acceptance are also non-negligible factors. Most tinnitus patients tend to attribute their condition to somatic diseases rather than psychological factors, and therefore consider psychological therapy inappropriate, hesitating to seek it (Weise et al., 2008). Some tinnitus patients expect to completely eliminate tinnitus perception and have low acceptance of CBT aimed at reducing tinnitus distress. Additionally, some tinnitus patients often perceive stigma when seeking support from psychotherapists. In a survey on patients’ attitudes towards psychotherapy, up to 23% of patients were skeptical of psychotherapy, and 11% had self-stigmatizing thoughts (Moritz et al., 2013).

Currently, the field still confront multiple challenges that impede the advancement of CBT for tinnitus. To address these barriers, the following suggestions are proposed for future directions in this field: (1) Establishing multidisciplinary tinnitus clinics. Such centers should systematically investigate the pathophysiological mechanisms and heterogeneous characteristics of tinnitus while enhancing the treatment of tinnitus comorbidities (Kajüter et al., 2024). Concurrent implementation of specialized psychotherapy training programs for audiologists is recommended to mitigate clinical psychologists shortages (Taylor et al., 2020). (2) Implementing public health campaigns to improve patient understanding of tinnitus psychophysiology and reduce their resistance to psychotherapy. Healthcare institutions should implement structured patient-centered communication channels and enhance psychological support mechanisms to reduce patients’ self-isolation and stigma concern (Marks et al., 2019). (3) Funding allocations for tinnitus psychotherapy research and clinical services should be substantially augmented, with optimization of referral pathways within healthcare systems to enhance treatment accessibility.

#### 4.2.3 Various forms of CBT for tinnitus

Despite the clinical implementation of conventional CBT for tinnitus management has progressed slowly and faces persistent challenges, its derivative modalities—such as internet-delivered cognitive behavioral therapy (iCBT), ACT, and mindfulness-based approaches—have garnered increasing research attention in recent years (Figures 7A,C). Technological adaptability and theoretical innovation may be principal factors of this shift in research focus. Over the past two decades, advancements in modern information technology, particularly the development of the internet, have significantly impacted healthcare services, psychological assessment, and treatment (Andersson, 2018). Internet-delivered interventions were initially developed and assessed in the mid-1990s (Ruwaard et al., 2011), and have since gained widespread dissemination and increasing recognition, iCBT being the most common form of internet intervention (Andersson, 2018). Compared to traditional group CBT, internet-delivered self-help CBT for tinnitus demonstrates equivalent efficacy and may serve as a potential alternative to group CBT (Jasper et al., 2014). Furthermore, iCBT effectively enhances patient compliance by improving treatment accessibility and reducing costs, overcoming some of the limitations of traditional therapies and marking a significant advancement in tinnitus management (Sia et al., 2024). In terms of clinical research innovation, internet interventions, with their advantages of being unrestricted by geography, enabling rapid recruitment of global participants, facilitating the acquisition of large sample sizes, and having relatively short research durations, have paved new avenues for tinnitus treatment and related research (Ritterband et al., 2006; Andersson et al., 2019).

Although the goal of iCBT programs is to reduce the impact of tinnitus through cognitive-behavioral strategies, these interventions are not uniform. They differ in content design, guidance format, and technological integration. iCBT interventions can generally be classified into guided and unguided formats based on the type of support provided. Guided iCBT typically involves support from therapists or professionals via telephone, email, or platform-based feedback. Therapist-supported online interventions not only improve patient adherence and reduce dropout rates, but also provide personalized guidance (Andersson and Titov, 2014). Research has demonstrated that guided iCBT is effective for managing chronic tinnitus (Weise et al., 2016). Similarly, audiologist-guided iCBT has also been shown to significantly reduce tinnitus-related distress and associated symptoms, with stable long-term outcomes (Beukes et al., 2018a; Beukes et al., 2018b). However, the role of therapist support in iCBT has yielded mixed results in earlier studies. For instance, Baumeister et al. found that therapist-guided internet interventions were more effective than unguided ones (Baumeister et al., 2014). Conversely, a randomized controlled trial comparing on-demand guided iCBT with unguided iCBT for tinnitus showed that iCBT effectively reduced tinnitus distress regardless of therapist support (Rheker et al., 2015).

In contrast, unguided iCBT emphasizes self-management and the use of self-help strategies. Due to its flexibility and low cost, this format is particularly suitable for resource-limited healthcare settings. Tinnitus patients with significant comorbidities (Bhatt et al., 2017) (e.g., anxiety, depression, or personality disorders) may require more therapist guidance and support than those with milder symptoms. Moreover, since patients in unguided iCBT are expected to complete modules independently without receiving motivational prompts, feedback, or guidance, dropout rates tend to be high. A systematic review evaluating four studies of unguided iCBT for tinnitus reported post-treatment dropout rates ranging from 16.1 to 37%, with follow-up dropout rates reaching 42.9% (Demoen et al., 2023). A meta-analysis on self-guided iCBT for depression also suggested that low educational attainment and comorbid anxiety could increase the risk of dropout (Karyotaki et al., 2015), which may also be relevant to the high attrition observed in unguided iCBT for tinnitus.

Additionally, app- or web-based telemedicine has emerged as a promising alternative to traditional therapies (Walter et al., 2025). One study was the first to validate the effectiveness of an iCBT mobile application, “Timibot,” which uses a chatbot interface (Bardy et al., 2024). It significantly alleviated tinnitus distress and, when combined with remote psychological counseling, enabled rapid symptom improvement in the early stages of treatment. This suggests the potential for integrating digital therapeutics with remote psychotherapy in tinnitus care.

Third wave CBT is an emerging approach in the field of modern psychotherapy, evolving and developing from existing cognitive-behavioral interventions (Brown et al., 2011). It aims to enhance the effectiveness of the first and second waves by emphasizing strategies that focus on contextual and experiential changes (Carvalho et al., 2017). ACT and MBCT are two highly regarded therapies within the third wave. ACT is a behavioral treatment grounded in functional contextualism and guided by relational frame theory (Hayes et al., 2006). It promotes healthy and value-based behavioral change by employing acceptance-based strategies that reduce individuals’ efforts to control or avoid internal experiences. A randomized controlled trial comparing ACT and CBT for the treatment of tinnitus found that both were equally effective in alleviating tinnitus-related distress (Hesser et al., 2012). In a randomized controlled trial, Westin et al. compared ACT with tinnitus retraining therapy (TRT) and found that the ACT group showed superior outcomes in tinnitus-related distress compared to TRT (Westin et al., 2011). MBCT integrates elements of mindfulness-based stress reduction (MBSR) and CBT, aiming to alter individuals’ responses to their own thoughts and feelings through the practice of mindfulness, and is commonly used to treat recurrent depressive disorders (Williams and Kuyken, 2012). In recent years, it has also been applied to the treatment of tinnitus. McKenna et al. (2017) conducted a randomized controlled trial comparing the efficacy of MBCT and intensive relaxation therapy (RT) in the treatment of chronic, distressing tinnitus. The results indicated that after an 8-week treatment period, patients in the MBCT group experienced significantly greater reductions in tinnitus severity compared to the RT group, with the therapeutic effects persisting at a 6-month follow-up. Moreover, both groups showed significant reductions in tinnitus loudness, psychological distress, anxiety, and depression. A further large-scale clinical study validated the effectiveness of MBCT in tinnitus treatment. In this study, standardized MBCT was applied to 182 adult patients with chronic tinnitus over an 8-week intervention period. The findings revealed that approximately 40% ~ 50% of the patients experienced significant improvements in tinnitus-related distress and psychological distress after the intervention (McKenna et al., 2018). This result not only reinforces the potential of MBCT as a therapeutic approach for tinnitus but also provides strong evidence support for its future clinical application.

### 4.3 Limitations

Firstly, the data for this study were exclusively sourced from the WoSCC database. While its authority and comprehensive scope are widely recognized, this choice may overlook relevant literature from other important databases such as PubMed, Google Scholar, and Scopus. Therefore, future research should consider expanding the range of databases to enrich the sample size of the study population. Secondly, this study was limited to original research articles and reviews published in English, potentially excluding papers published in other languages and other types of publications. Thirdly, data collection and processing were heavily reliant on software tools, and the parameter settings and analytical methods of the software lack systematic standards, which may lead to variations in the results. Despite these limitations, bibliometric analysis offers a valuable insight of the research tendencies and hotspots regarding cognitive-behavioral therapy for tinnitus.

## 5 Conclusion

This study, through bibliometric analysis, identified that the treatment of tinnitus with comorbid psychological disorders (depression and anxiety) is a key research trend in this field. In this context, various forms of CBT for tinnitus, such as iCBT, ACT, and MBCT, have emerged as research hotspots. Although existing studies have confirmed the effectiveness of CBT in tinnitus treatment, current research still faces challenges. Therefore, future efforts should focus on conducting large-scale, multicenter clinical studies with standardized research protocols to further verify the long-term efficacy and cross-cultural applicability of CBT. Additionally, it is essential to explore the integration of CBT with other treatment methods, enhance interdisciplinary collaboration, and promote the development of the field of tinnitus treatment.

## Data Availability

The original contributions presented in the study are included in the article/supplementary material, further inquiries can be directed to the corresponding authors.
